# Comparing vegetation indices for remote chlorophyll measurement of white poplar and Chinese elm leaves with different adaxial and abaxial surfaces

**DOI:** 10.1093/jxb/erv270

**Published:** 2015-06-01

**Authors:** Shan Lu, Xingtong Lu, Wenli Zhao, Yu Liu, Zheyi Wang, Kenji Omasa

**Affiliations:** ^1^School of Geographical Sciences, Northeast Normal University, 5268 Renmin Street, Changchun 130024, China; ^2^Graduate School of Agricultural and Life Sciences, The University of Tokyo, Yayoi 1-1-1, Bunkyo-ku, Tokyo 113–8657, Japan

**Keywords:** Adaxial and abaxial leaf surfaces, chlorophyll, phenotyping, reflectance, remote sensing, vegetation index.

## Abstract

Quick non-destructive assessment of leaf chlorophyll content (LCC) is important for studying phenotypes related to plant growth and stress resistance. This study was undertaken to investigate the quantitative relationship between LCC and different vegetation indices (VIs) on both adaxial and abaxial surfaces of white poplar (*Populus alba*), which has dense tubular hairs on its abaxial surface, and Chinese elm (*Ulmus pumila* var. *pendula*), which does not show obvious superficial differences except for lighter colour on the abaxial surface. Some published and newly developed VIs were tested to relate them to LCC. The results showed that most of the published VIs had strong relationships with LCC on the one-surface dataset, but did not show a clear relationship with LCC when both adaxial and abaxial surface reflectance data were included. Among the reflectance indices tested, the modified Datt index, (*R*_719_−*R*_726_)/(*R*_719_−*R*_743_), performed best and is proposed as a new index for remote estimation of chlorophyll content in plants with varying leaf surface structures. It explained 92% of LCC variation in this research, and the root mean square error of the LCC prediction was 5.23 μg/cm^2^. This new index is insensitive to the effects of adaxial and abaxial leaf surface structures and is strongly related to the variation in reflectance caused by chlorophyll content.

## Highlight

A new vegetation index insensitive to adaxial and abaxial leaf surfaces was proposed in this paper to accurately estimate leaf chlorophyll content.

## Introduction

The study of spectrometric remote sensing of leaves is relevant because the spectral features are related to nondestructive monitoring of plant growth and health (Cordón and Lagorio, 2007) and are partly correlated with plant biochemical components (Vogelmann *et al.*, 1993; Peñuelas *et al.*, 1997; Smith *et al.*, 1997; Adams *et al.*, 1999). Several attempts have been made to use remote spectral measurements to determine leaf chemistry at both leaf and canopy levels (Gitelson and Merzlyak, 1994; Kim *et al.*, 1994; Curran *et al.*, 1995; Peñuelas *et al.*, 1995; Rondeaux *et al.*, 1996; Blackburn, 1998a; Datt, 1998; Daughtry *et al.*, 2000; Haboudane *et al.*, 2002; Richardson *et al.*, 2002). Leaf chlorophyll (Chl) content, as one of the most important vegetative parameters, provides valuable information not only on the physiological status, but also on the phenotypic manifestations of plants (Fiorani and Schurr, 2013; Ainsworth *et al.*, 2014). Hence, there is a need for accurate, efficient and practical methodologies to estimate it (Levizou *et al.*, 2005; Steele *et al.*, 2008; Fiorani and Schurr, 2013). Non-destructive remote determination of leaf chlorophyll content (LCC) permits measurement of Chl variation over time for a single leaf and avoids time-consuming and expensive traditional Chl content measurements (Sadras *et al.*, 2000; Sims and Gamon, 2002).

Decades of research have gone into finding Chl-sensitive regions from the vegetation spectrum that can be non-destructively extracted (and quantified) using combinations of wavebands [i.e. vegetation indices (VIs)] (Main *et al.*, 2011). VIs have been introduced for large-scale phenotyping of biomass, as well as studies on greenness, nitrogen content, pigment composition and photosynthetic status (Fiorani and Schurr, 2013). Several studies have found that the wavelengths adjoining the Chl maximum absorption bands (700nm) and the green (550nm) bands are the most sensitive to a wide range of Chl contents (Gitelson and Merzlyak, 1994, 1996; Lichtenthaler *et al.*, 1996). Indices for Chl estimation based on reflectances in narrow spectral bands have recently received considerable attention and have been found to be well correlated with the Chl content of leaves (e.g. 1/*R*_700_, 1/*R*_550_−1/*R*_750_, *R*_800_/*R*_650_, NDI: (*R*_750_−*R*_705_)/(*R*_750_+*R*_705_), *D*_730_ or *D*_740_) (Chappelle *et al.*, 1992; Gitelson and Merzlyak, 1994, 1996; Peñuelas *et al.*, 1994; Curran *et al.*, 1995; Blackburn, 1998a, 1998b; Datt, 1998, 1999a, 1999b; Adams *et al.*, 1999; Zarco-Tejada *et al.*, 2001; Richardson *et al.*, 2002; Sims and Gamon, 2002; Mutanga and Skidmore, 2007). Several researchers have also worked to develop an algorithm that would be minimally sensitive to differences in leaf structure to avoid species-specific calibration (Datt, 1999b; Maccioni *et al.*, 2001; Gitelson *et al.*, 2003). However, the authors are aware of few studies that have attempted to assess how the accuracy of Chl content estimation depends on the adaxial (upper) and abaxial (lower) surfaces of leaf spectral bands.

While the remotely sensed data are being acquired, multiple scattering of higher-order canopy causes the incoming solar radiation to be reflected from understory and other leaves and enter the abaxial side of leaves. Furthermore, some foliage may change its orientation, turning the adaxial leaf surface away from the sun and exposing the abaxial leaf surface (Campbell *et al.*, 2007). This would make the remote sensing data contain spectral information from both adaxial and abaxial leaf surfaces. The phenotypic expressions (such as leaf hair, wax, palisade tissues, spongy tissues, etc.) of leaves vary not only between species, but also between the adaxial and abaxial surfaces of one leaf. Whereas the reflectance signal registered by remote sensors reveals fairly definitive structural changes in leaves and vegetation canopy covers (Demarez *et al.*, 1999; Slaton *et al.*, 2001; Levizou *et al.*, 2005; Serrano, 2008; Rautiainen *et al.*, 2010; Verrelst *et al.*, 2012), Chl content estimates made solely from VIs of the adaxial leaf surface may not be accurate enough (Lu and Lu, 2015). Sims and Gamon (2002) also suggested that Chl detection would tend to be limited to adaxial leaf layers. The authors’ research group has tested the validity of already-published VIs on predicting SPAD (soil plant analysis development) values of leaves on adaxial or abaxial surfaces (Lu and Lu, 2015), but the performance of the VIs on both adaxial and abaxial surfaces was not evaluated, nor were effective models superior to the already-published VIs suggested.

This study presents an analysis of the reflectance spectra of leaves belonging to two species (white poplar and Chinese elm), which are very common plants in northeast China. These species possess very different phenotypic characteristics, especially with regard to leaf surface structures; their study provides more general information on the reflectance of plant leaves. The main objective of this study is to find spectral indices for LCC estimation on variously structured leaves that are not sensitive to the differences between adaxial and abaxial leaf surfaces. To determine ‘universal’ Chl indices, i.e. indices applicable to different species and leaf surfaces, the newly proposed LCC indices were compared with the previously suggested VIs and the most effective vegetation indices were found for measuring LCC over a wide range of plant species and leaf structures.

## Materials and methods

### Plant materials

Sampling was carried out on the campus of Northeast Normal University, China. Two deciduous tree species, white poplar (*Populus alba*) and Chinese elm (*Ulmus pumila* var. *pendula*), were chosen because of their considerable leaf structural differences, with one individual of each sampled. Fifty-five leaf samples of white poplar and 60 of Chinese elm were detached randomly from the stem, immediately packed and sealed into plastic bags and placed inside a cooler to avoid desiccation and decomposition of the Chl by light. All the measurements, including the reflectance measurements and Chl extraction, were carried out within 4h after leaf harvesting to minimize changes in Chl content.

Microscopic photographs of adaxial and abaxial leaf surfaces were taken using an Olympus microscope equipped with a CCD camera. The magnification was 10× the original size.

### Spectral measurements

Immediately following leaf sample delivery to the laboratory, spectral reflectance was measured on adaxial and abaxial leaf surfaces using an ASD FieldSpec® 3 portable spectrometer (Analytical Spectral Devices, Boulder, CO, USA). The spectrometer can collect data in the 350–2500nm spectral region, with a sampling interval of 1.4nm in the 350–1000nm wavelength range and 2nm in the 1000–2500nm wavelength range. Because the VIs involved in this study were related only to wavelengths of 400–1000nm, a subset of the reflectance spectra were used within this range. Three scans for each sample were conducted and the measurements were converted to reflectance after comparing with a 99% diffuse reflector (Spectralon®, Labsphere, North Sutton, NH, USA). The average reflectance was taken as representative of the sample. The reflectance measurements were made with the help of a leaf clip equipped with an internal halogen source directly attached to the leaf surface. The reflectance spectra were collected at a 45° angle from the nadir direction (0°/45° irradiation/detection geometry). This configuration was the same as in the research of Demarez *et al.* (1999). They suggested that this configuration could reduce some influence of leaf specular reflectance. The measurements from other directions would agree with the results of this research if a piece of leaf were considered as a Lambertian surface (Chelle, 2006). For the reflectance measurement, all the samples were put on a piece of black paper to avoid transmission of extraneous reflectances from the reflected light through the leaf.

### Chlorophyll content measurements

For each leaf, Chl was extracted from the approximate location of leaf disc used for reflectance measurements. The disc was cut into small pieces and ground in the dark with a mortar and pestle in 95% (v/v) ethanol until the pulp turned white in colour and all pigments were extracted. Thereafter, the leaf pigment mixture was moved to a 50ml volumetric flask with 95% ethanol and one part of the mixture was centrifuged in plastic tubes with a rotational speed of 3200 r/min for 10min. The supernatant was decanted from the tubes and its absorbance immediately measured with a Lambda 900 spectrophotometer (Perkin-Elmer, Waltham, MA, USA). Chl content (μg/cm^2^) was calculated according to Wintermans and De Mots (1965).

### Data analysis

More than 30 published Chl indices (Table 1) for estimation from spectral analysis were tested with the experimental data. Most of the tested indices can be classified into five categories: (i) single-band reflectance or single-difference (SD) index between the reflectance of two bands (e.g. *R*_680_ or 1/*R*_550_−1/*R*_750_); (ii) simple-ratio (SR) index (e.g. *R*_672_/*R*_550_); (iii) normalized difference (ND) of reflectance (e.g. NDVI: (*R*_800_−*R*_650_)/(*R*_800_+*R*_650_)); (iv) indices using reflectance derivatives (e.g. *D*_730_); and (v) other forms of indices (e.g. *R*_705_/(*R*_717_+*R*_491_)).

**Table 1.** Chlorophyll indices used in this study

**Classification**	**Vegetation index**	**References**
Single-band reflectance or SD (simple difference) indices	1/*R*_700_	Gitelson and Merzlyak, 1996
	*R*_680_	Blackburn, 1998b
	1/*R*_700_−1/*R*_750_	Gitelson *et al.*, 2003
	1/*R*_550_−1/*R*_750_	Gitelson *et al.*, 2003
	SD: *R*_λ1_−*R*_λ2_	This paper
SR (simple ratio) indices	*R*_750_/*R*_550_	Lichtenthaler *et al.*, 1996
	*R*_750_/*R*_700_	Lichtenthaler *et al.*, 1996
	*R*_860_/*R*_550_	Datt, 1998
	*R*_672_/*R*_550_	Datt, 1998
	PSSR_*a*_: *R*_800_/*R*_680_	Blackburn, 1998a
	PSSR_*b*_: *R*_800_/*R*_635_	Blackburn, 1998a
	*R*_800_/*R*_650_	Blackburn, 1998b
	*R*_800_/*R*_675_	Blackburn, 1998b
	*R*_450_/*R*_550_	Zarco-Tejade et al., 2001
	*R*_750_/*R*_710_	Zarco-Tejada *et al.*, 2001
	*R*_950_/*R*_680_	Zhu *et al.*, 2007
	SR: *R*_λ1_/*R*_λ2_	This paper
ND (normalized difference) indices	NDI: (*R*_750_−R_705_)/(*R*_750_+*R*_705_)	Gitelson and Merzlyak, 1994
	PSND_*b*_: (*R*_800_−*R*_635_)/(*R*_800_+*R*_635_)	Blackburn, 1998a
	(*R*_800_−*R*_650_)/(*R*_800_+*R*_650_)	Blackburn, 1998b
	(*R*_800_−*R*_675_)/(*R*_800_+*R*_675_)	Blackburn, 1998b
	ND: |(*R*_λ1_−*R*_λ2_)|/(*R*_λ1_+*R*_λ2_)	This paper
Indices using reflectance derivatives	*D*_754_/*D*_704_	Takebe and Yoneyama, 1989
	$RII=\int_{705}^{750}\left(R_{\lambda }/R_{705}-1\right)d\lambda$	Richardson *et al.*, 2002
	*D*_730_	Richardson *et al.*, 2002
	*D*_710_	Mutanga and Skidmore, 2007
	*D*_740_	Mutanga and Skidmore, 2007
Others	VOG_2_: (*R*_734_−*R*_747_)/(*R*_715_+*R*_726_)	Vogelmann *et al.*, 1993
	CARI:(|(a*670+*R*_670_+b)|/(a^2^+1)^0.5^)*(*R*_700_/*R*_670_) [a=(*R*_700_−*R*_550_)/150; b=*R*_550_−(a*550)]	Kim *et al.*, 1994
	*R*_672_/(*R*_550_×*R*_708_)	Datt, 1998
	*R*_860_/(*R*_550_×*R*_708_)	Datt, 1998
	MCARI: [(*R*_700_−*R*_670_)−0.2*(*R*_700_−*R*_550_)]*(*R*_700_/*R*_670_)	Daughtry *et al.*, 2000
	TCARI/OSAVI:3*[(*R*_700_−*R*_670_)−0.2*(*R*_700_−*R*_550_)*(*R*_700_/*R*_670_)]/[(1+0.16)*(*R*_800_–*R*_670_)/(*R*_800_+*R*_670_+0.16)]	Daughtry *et al.*, 2000; Rondeaux *et al.*, 1996
	TCARI: 3*[(*R*_700_−*R*_670_)−0.2*(*R*_700_−*R*_550_)*(*R*_700_/*R*_670_)]	Haboudane *et al.*, 2002
	*R*_705_/(*R*_717_+*R*_491_)	Tian *et al.*, 2011
	*R*_434_/(*R*_496_+*R*_401_)	Tian *et al.*, 2011
	(*R*_850_−*R*_710_)/(*R*_850_−*R*_680_)	Datt, 1999b
	MDATT index: (*R*_λ3_−*R*_λ1_)/(*R*_λ3_−*R*_λ2_)	This paper

In addition, two-band indices were evaluated using a custom-developed computer programme to traverse all band combinations of the SD, SR and ND indices. These indices were calculated using two random available wavebands (*λ*_1_ and *λ*_2_) in the 400–1000nm region to select the best two-band indices as well as the extent of the effective two-band combination regions for assessment of Chl content, as shown in Eqs (1)–(3):

$$SD(R_{\lambda_{1}},R_{\lambda_{2}})=R_{\lambda_{1}}-R_{\lambda_{2}}$$ (1)

$$SR(R_{\lambda_{1}},R_{\lambda_{2}})=\frac{R_{\lambda_{1}}}{R_{\lambda_{2}}}$$ (2)

$$ND(R_{\lambda_{1}},R_{\lambda_{2}})=\frac{\left|R_{\lambda_{1}}-R_{\lambda_{2}}\right|}{R_{\lambda_{1}}+R_{\lambda_{2}}}$$ (3)

Furthermore, three-band indices based on the Datt (1999b) principle were derived by introducing a third band (*λ*_3_) to the indices according to Eq. (4):

$$\text{Modified Datt (MDATT) index }(R_{\lambda_{1}},R_{\lambda_{2}},R_{\lambda_{3}})=\frac{R_{\lambda_{3}}-R_{\lambda_{1}}}{R_{\lambda_{3}}-R_{\lambda_{2}}}$$ (4)

This MDATT index was modified to compensate for high leaf surface (specular) reflectance and scattering from the mesophyll, which tend to alter reflectance across the whole visible and near-infrared spectrum. Adding a constant (the specular reflectance and scattering from mesophyll) to all reflectance values changes the indices even when there is no change in absorptance of tissues below the epidermis (Sims and Gamon 2002). The MDATT index was developed here to remove this effect.

All two-band, MDATT and published indices derived from adaxial or abaxial surfaces (*n*=110 for the white poplar and *n*=120 for the Chinese elm) and from both leaf surfaces for the two species together (*n*=230) were correlated with Chl content. The relationships with the best-fit coefficient of determination (*R* ^2^) and root mean square error (*RMSE*) were used to evaluate the effectiveness of each index in estimating Chl content and in selecting the most applicable indices that are not sensitive to the leaf surface.

## Results

### Structure of adaxial and abaxial leaf surfaces

**Fig. 1.**
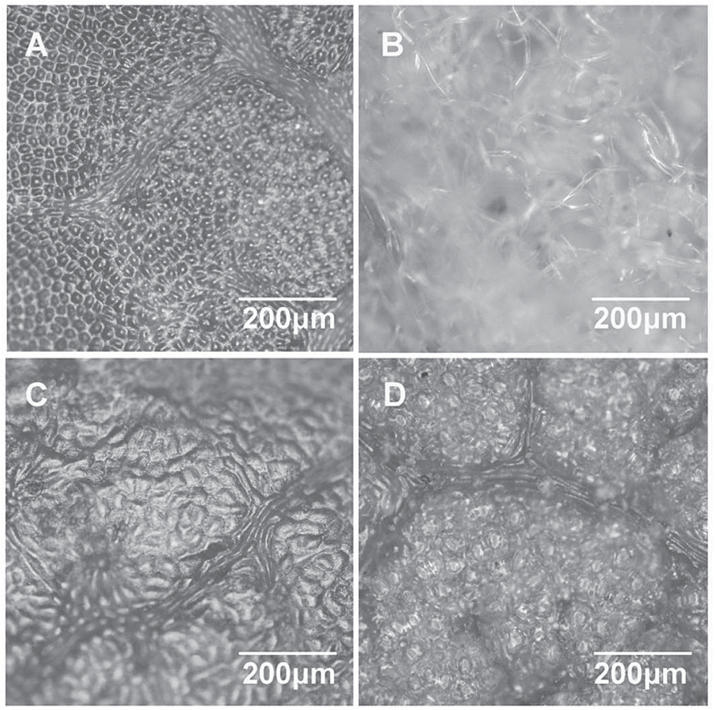
Optical microscopic reflectance images of leaf surfaces of white poplar and Chinese elm: (A) adaxial and (B) abaxial surfaces of white poplar; (C) adaxial and (D) abaxial surfaces of Chinese elm. Bar, 200 μm.

The smooth adaxial surface (Fig. 1A) of the white poplar leaves differed greatly from the abaxial surface, which has large amounts of tubular hair. The hair was so dense that the cuticular structure could not be seen from the microscopic photograph (Fig. 1B). Because leaf hair, as a major determinant of leaf surface relief, may change leaf surface scattering as well as reflectance, the abaxial surface of white poplar appears to have a white or silver colour. By contrast, the adaxial and abaxial surfaces of Chinese elm leaves had fewer superficial structural differences (Fig. 1C, D), except for a little lighter green on the abaxial surface. Studying the different leaf surfaces of plants may help to acquire more general information on the reflectance of plant leaves and to obtain more applicable VIs to estimate LCC on variously structured leaves.

### Spectral reflectance of adaxial and abaxial leaf surfaces

**Fig. 2.**
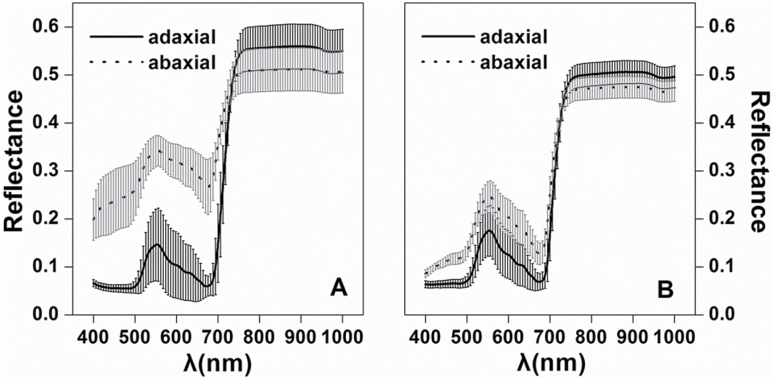
Average (the curves) and standard deviation (the shaded area) of reflectance spectra of adaxial and abaxial leaf surfaces for (A) white poplar (*n*=55) and (B) Chinese elm (*n*=60). λ is the wavelength.

The reflectance spectra for adaxial and abaxial surfaces of white poplar and Chinese elm are presented in Fig. 2. It is apparent that the reflectance spectra were much lower in the visible wavelengths (400–680nm) for the adaxial surface than for the abaxial surface. However, the difference in the near infrared (NIR) wavelengths (760–1000nm) was not significant. Furthermore, the difference between the spectral reflectance of adaxial and abaxial surfaces for the white poplar leaf (Fig. 2A) was significantly greater than that for Chinese elm (Fig. 2B) in the visible wavelengths.

**Fig. 3.**
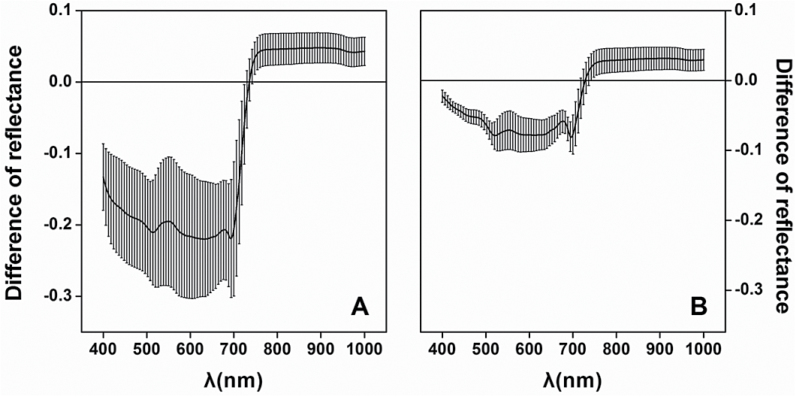
Difference in reflectance between adaxial and abaxial surfaces of (A) white poplar and (B) Chinese elm.

The differences in reflectance between adaxial and abaxial surfaces are shown in Fig. 3. A distinct difference could be found between the surfaces in the white poplar leaves, but a smaller variation was shown in the Chinese elm leaves, a result which agreed with their leaf surface appearance and microscopic photographs. In white poplar leaves, the least reflectance difference between adaxial and abaxial surfaces occurred at 735nm wavelength and it occurred at 728nm in Chinese elm leaves.

### Relationship between LCC and new two-band indices derived from both adaxial and abaxial surfaces of white poplar and Chinese elm

**Fig. 4.**
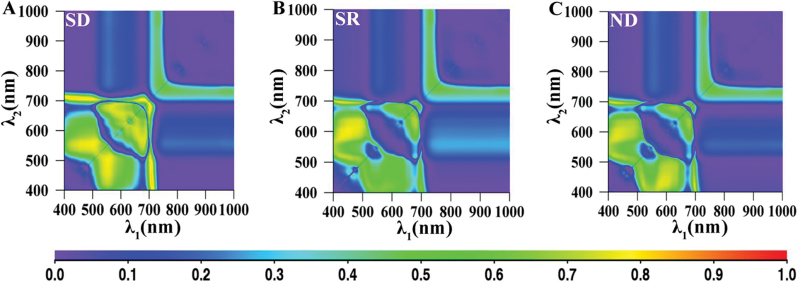
The map for coefficient of determination (*R* ^2^) between the two-band simple difference (Eq. 1), simple ratio (Eq. 2) and normalized difference (Eq. 3) indices and leaf chlorophyll content for both surfaces of both plant species. (A) simple difference, (B) simple ratio, (C) normalized difference.

The *R* ^2^ values between the LCC and the SD, SR and ND indices are shown in Fig. 4, where the *x*-axis represents *λ*_1_ and the *y*-axis *λ*_2_. In this figure, 230 samples comprising the adaxial and abaxial surfaces of both species, were used to calculate *R* ^2^. These maps provide an overview of the statistical significance of the indices for all combinations of two wavelengths. They enable efficient extraction of significant peak wavelengths as well as the extent of the effective regions for assessment of Chl content. The results showed that the LCC-sensitive regions mainly involved two areas. One was made up of red edge and blue wavelengths, with *λ*_1_ ranging from 705 to 715nm and *λ*_2_ from 420 to 440nm.

The SD, SR and ND indices from two random bands that had rather high *R* ^2^ and selected published indices (*R* ^2^>0.65, *n*=230) are listed in Table 2 (see Supplementary Table S1 for the performance of all the indices tested in this study). They were evaluated for their ability to predict Chl content based on the datasets for the two species together (*n*=230) and for each species separately (*n*=110 for white poplar and *n*=120 for Chinese elm). The *R* ^2^ and *RMSE* for each predictive model are also provided in Table 2.

**Table 2.** Relationships between vegetation indices and leaf chlorophyll content for both adaxial and abaxial data of two plants with different leaf surfaces

Vegetation indices	**Both plants**		Vegetation indices	**White poplar**		Vegetation indices	**Chinese elm**	
	*R* ^2^	*RMSE* (μg/cm^2^)		*R* ^2^	*RMSE* (μg/cm^2^)		*R* ^2^	*RMSE* (μg/cm^2^)
MDATT: (*R*_λ3_−*R*_λ1_)/(*R*_λ3_−*R*_λ2_) (*R* ^2^>0.92; λ_1_,726~728; λ_2_, 743~743; λ_3_, 717~720) (*R* ^2^>0.90; λ_1_,721~746; λ_2_,705~758; λ_3_, 699~798) (*R*_719_−*R*_726_)/(*R*_719_−*R*_743_) *	0.92	5.23	MDATT: (*R*_719_−*R*_732_)/ (*R*_719_−*R*_726_) *	0.94	4.67	MDATT: (*R*_719_−*R*_747_)/ (*R*_719_−*R*_721_) *	0.91	4.53
SD: *R*_λ1_−*R*_λ2_ (*R* ^2^>0.73; λ_1_,705−715; λ_2_, 420~440) *R*_709_−*R*_434_	0.81	7.77	SD: *R*_708_−*R*_434_	0.83	8.07	SD: *R*_712_−*R*_426_	0.76	7.35
SR: *R*_λ1_/*R*_λ2_ (*R* ^2^>0.70; λ_1_, 400~680; λ_2_, 510~702) *R*_451_/*R*_604_	0.78	8.51	*D*_754_/*D*_704_	0.81	8.66	SR: *R*_421_/*R*_700_	0.75	7.42
*D*_754_/*D*_704_	0.76	8.85	SR:*R*_434_/*R*_517_	0.80	8.78	ND: (*R*_700_−*R*_420_)/ (*R*_700_+*R*_420_)	0.73	7.66
ND: |(*R*_λ1_-*R*_λ2_)|/(*R*_λ1_+*R*_λ2_) (*R* ^2^>0.60; λ_1_, 515~605 or 700~705; λ_2_, 410~435) (*R*_583_−*R*_426_)/(*R*_583_+*R*_426_)	0.76	8.86	TCARI/OSAVI	0.78	9.26	*D*_754_/*D*_704_	0.72	7.92
(*R*_850_−*R*_710_)/(*R*_850_−*R*_680_)	0.73	9.25	ND: (*R*_516_−*R*_431_)/ (*R*_516_+*R*_431_)	0.78	9.29	(*R*_850_−*R*_710_)/ (*R*_850_−*R*_680_)	0.68	8.43
TCARI/OSAVI	0.71	9.72	(*R*_850_−*R*_710_)/(*R*_850_−*R*_680_)	0.76	9.75	VOG_2_	0.67	8.47
*R*_705_/(*R*_717_+*R*_491_)	0.65	10.58	*R*_705_/(*R*_717_+*R*_491_)	0.65	11.66	*D*_740_	0.67	8.49

*, the MDATT indices that performed best in each dataset. ‘Both plants’ dataset, *n*=230; white poplar dataset, *n*=110; Chinese elm dataset, *n*=120.

The two-band indices which performed best among the two-band spectral indices for the three datasets were the SD indices using the red edge and blue wavelength combination (see Fig. 4 and Table 2). The best-performing two-band spectral index was the SD *R*_709_ *−R*_434_ index (*R* ^2^=0.81, *RMSE*=7.77 μg/cm^2^) for both leaf surfaces in the two plant dataset, whereas the SD *R*_708_ *−R*_434_ index (*R* ^2^=0.83, *RMSE*=8.07 μg/cm^2^) was the best for both leaf surfaces of white poplar and the SD *R*_712_ *−R*_426_ index (*R* ^2^=0.76, *RMSE*=7.35 μg/cm^2^) for both leaf surfaces of Chinese elm.

### Relationship between LCC and MDATT indices derived from both adaxial and abaxial surfaces of white poplar and Chinese elm

**Fig. 5.**
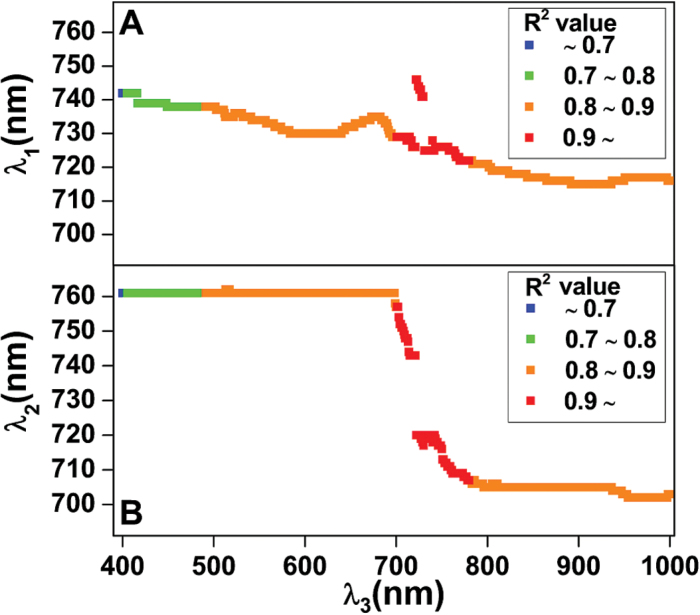
Changes in the best coefficient of determinations (*R* ^2^) between the MDATT indices and leaf chlorophyll content as derived from combinations of wavelengths of λ_1_, λ_2_ and λ_3_. (A) combination of λ_1_ and λ_3_, (B) combination of λ_2_ and λ_3_.

**Fig. 6.**
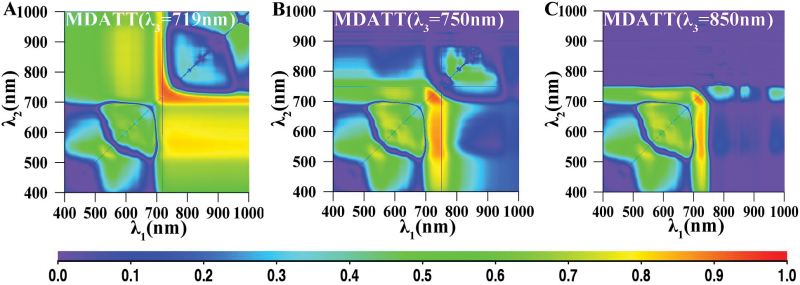
The map for coefficient of determination (*R* ^2^) between the MDATT indices and the leaf chlorophyll content for both surfaces of both species. (A) λ_3_=719nm, (B) λ_3_=750nm, (C) λ_3_=850nm.

To identify optimal parameters for estimating LCC, further analysis was conducted on the MDATT indices, which were based on the same dataset analysed for the two-band indices. Each combination of three bands was used to compose an MDATT index that was then correlated with the LCC. The best *R* ^2^ values between the LCC and the MDATT indices generated from combinations of wavelengths *λ*_1_, *λ*_2_ and *λ*_3_ are shown in Fig. 5, and the performance of the MDATT indices in estimating LCC is also shown in Table 2. The results indicated that the MDATT indices that had good correlations with LCC were derived primarily from the red edge wavelength regions. For example, the MDATT indices with *R* ^2^ greater than 0.90 were derived from the wavelengths of *λ*_1_ (721~746nm), *λ*_2_ (705~758nm) and *λ*_3_ (699~798nm); the indices with *R* ^2^ greater than 0.92 were generated from the wavelengths of *λ*_1_ (726~728nm), *λ*_2_ (743nm) and *λ*_3_ (717~720nm); and the best-performing index overall was (*R*_719_ *−R*_726_)/(*R*_719_ *−R*_743_), which generated the most significant linear relationships with LCC (*R* ^2^=0.92, *RMSE*=5.23 μg/cm^2^) (Table 2). The maps for *R* ^2^ between the MDATT indices (*λ*_3_ fixed at 719nm, 750nm and 850nm) and the LCC for both surfaces of both species combined are shown in Fig. 6. The (*R*_719_ *−R*_732_)/(*R*_719_ *−R*_726_) and (*R*_719_ *−R*_747_)/(*R*_719_ *−R*_721_) indices were strongly related to LCC for the two-surface datasets of white poplar (*R* ^2^=0.94, *RMSE*=4.67 μg/cm^2^) and Chinese elm (*R* ^2^=0.91, *RMSE*=4.53 μg/cm^2^).

**Fig. 7.**
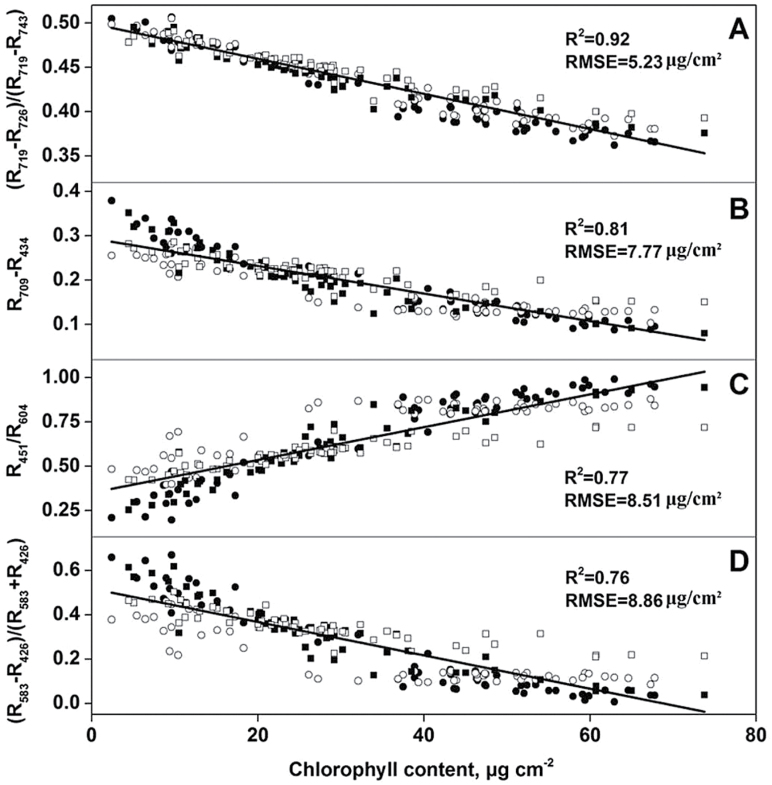
Best-fit lines and experimental data for vegetation indices (VIs) and chlorophyll content relationships: (A) (*R*_719_−*R*_726_)/(*R*_719_−*R*_743_) versus Chl; (B) *R*_709_−*R*_434_ versus Chl; (C) *R*_451_/*R*_604_ versus Chl; (D) (*R*_583_−*R*_426_)/(*R*_583_+*R*_426_) versus Chl. Filled circles, adaxial surface data for white poplar; closed circles, abaxial surface data for white poplar; filled squares, adaxial surface data for Chinese elm; closed circles, abaxial surface data for Chinese elm.

The scatter plots for the 230 samples between LCC and the best-performing two-band and MDATT indices are shown in Fig. 7. It can also be seen that the MDATT index correlated with LCC better than the two-band indices.

### Comparison of new LCC indices with published spectral indices derived from both adaxial and abaxial surfaces of white poplar and Chinese elm

Some published indices performed relatively well with LCC on the two-surface dataset, including the *D*_754_/*D*_704_ spectral index and the (*R*_850_ *−R*_710_)/(*R*_850_ *−R*_680_) Datt index. The indices with better relationships to LCC than the other published VIs are based on the red edge or the near infrared bands. They performed similarly to or slightly better than the selected ND or SR indices found in this study, as shown in Table 2. However, they only achieved an *R* ^2^ close to or less than 0.75 and an *RMSE* greater than 8.5 μg/cm^2^ and exhibited poorer LCC estimation than the new proposed MDATT indices.

### Relationship between LCC and the new proposed and published spectral indices derived from adaxial or abaxial surfaces of each plant

**Fig. 8.**
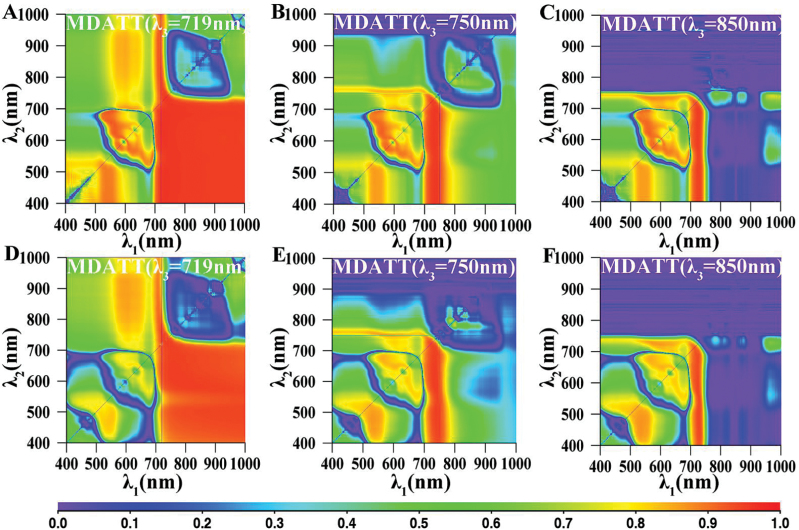
The map for coefficient of determination (*R* ^2^) between the MDATT index [MDATT=(*R*_λ3_−*R*_λ1_)/(*R*_λ3_−*R*_λ2_)] and leaf chlorophyll content for the adaxial and abaxial surfaces of white poplar. MDATT indices for adaxial surface with λ_3_ equal to (A) 719nm, (B) 750nm and (C) 850nm. MDATT indices for abaxial surface with λ_3_ equal to (D) 719nm, (E) 750nm and (F) 850nm.

**Fig. 9.**
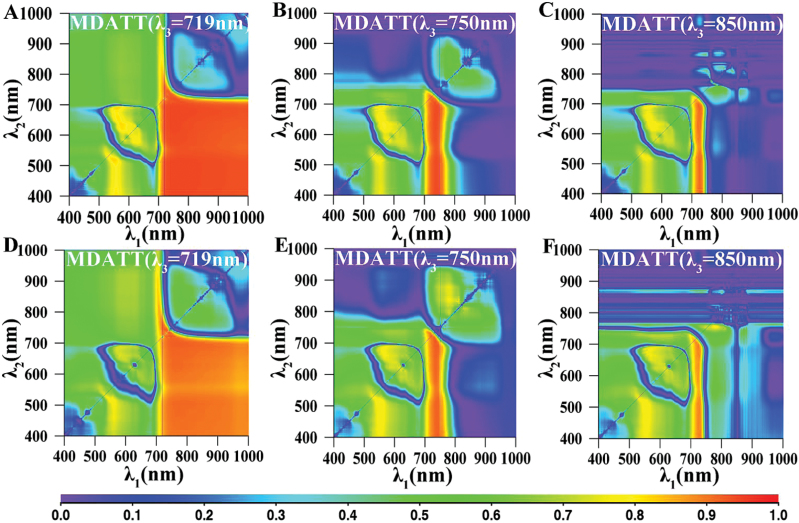
The map for coefficient of determination (*R* ^2^) between the MDATT index [MDATT=(*R*_λ3_−*R*_λ1_)/(*R*_λ3_−*R*_λ2_)] and leaf chlorophyll content for the adaxial and abaxial surfaces of Chinese elm. MDATT indices for adaxial surface with λ_3_ equal to (A) 719nm, (B) 750nm and (C) 850nm. MDATT indices for abaxial surface with λ_3_ equal to (D) 719nm, (E) 750nm and (F) 850nm.

Analysis of the datasets for each leaf surface was also conducted separately for each species to verify the effect of leaf surface on Chl content estimation. The distributions of *R* ^2^ for the MDATT indices when *λ*_*3*_ was set to 719nm, 750nm and 850nm for white poplar and Chinese elm leaves are shown in Figs 8 and 9. The LCC-sensitive region on adaxial or abaxial surfaces was occupied by the whole bottom right area when *λ*_3_ was 719nm. However, the sensitive range was narrower when *λ*_3_ was set to 750nm and 850nm. In addition, the LCC-sensitive region was broader on adaxial than on abaxial leaf surface.

The performance of the proposed and published indices in predicting LCC for the adaxial or abaxial surface of each plant species (*R* ^2^>0.84) are shown in Table 3 (see Supplementary Table S2 for the performance of all the indices tested in this study). Almost all the indices had higher *R* ^2^ on adaxial than on abaxial leaf surfaces (Figs 8, 9, Table 3). The MDATT indices (*λ*_3_=719) also performed well in estimating LCC, as did some SR indices.

**Table 3.** Relationships between vegetation indices and leaf chlorophyll content for individual plant species

**White poplar**						**Chinese elm**					
Vegetation indices	Adaxial surface		Vegetation indices	Abaxial surface		Vegetation indices	Adaxial surface		Vegetation indices	Abaxial surface	
	*R* ^2^	*RMSE* (μg/ cm^2^)		*R* ^2^	*RMSE* (μg/ cm^2^)		*R* ^2^	*RMSE* (μg/ cm^2^)		*R* ^2^	*RMSE* (μg/ cm^2^)
MDATT: (*R*_λ3_−*R*_λ1_)/(*R*_λ3_−*R*_λ2_) (*R* ^2^>0.96: λ_1_, 715~751,λ_2_, 646~739; λ_3_, 400~788) (*R*_719_−*R*_731_)/(*R*_719_−*R*_647_)*	0.96	3.84	MDATT: (*R*_719_−*R*_746_)/ (*R*_719_−*R*_718_) *	0.95	4.26	MDATT: (*R*_719_−*R*_761_)/ (*R*_719_−*R*_493_) *	0.95	3.51	MDATT: (*R*_719_−*R*_742_)/ (*R*_719_−*R*_732_) *	0.93	4.07
SR: *R*_λ1_/*R*_λ2_ (*R* ^2^>0.83; λ_1_, 740~780; λ_2_, 700~750) *R*_751_/*R*_720_	0.96	3.78	SR: *R*_747_/*R*_748_	0.92	5.52	SR: *R*_779_/*R*_709_	0.95	3.48	SR: *R*_742_/*R*_739_	0.92	4.17
ND:|(*R*_λ1_−*R*_λ2_)|/(*R*_λ1_+*R*_λ2_) (*R* ^2^>0.94; λ_1_, 740~750;λ_2_, 730~750) (*R*_740_−*R*_738_)/ (*R*_740_+*R*_738_)	0.96	3.83	ND: (*R*_748_−*R*_747_)/ (*R*_748_+*R*_747_)	0.92	5.52	*R*_750_/*R*_710_	0.95	3.51	ND: (*R*_742_−*R*_739_)/ (*R*_742_+*R*_739_)	0.92	4.19
*R*_750_/*R*_710_	0.96	3.83	SD: *R*_748_−*R*_747_	0.92	5.6	VOG_2_	0.94	3.54	VOG_2_	0.92	4.21
VOG_2_	0.96	3.90	(*R*_850_−*R*_710_)/ (*R*_850_−*R*_680_)	0.91	5.86	ND: (*R*_749_−*R*_738_)/ (*R*_749_+*R*_738_)	0.94	3.57	SD: *R*_749_−*R*_746_	0.91	4.45
SD: *R*_λ1_−*R*_λ2_ (*R* ^2^>0.94; λ_1_, 740~750;λ_2_, 740~750) *R*_744_−*R*_742_	0.95	4.30	VOG_2_	0.90	6.37	*RII*	0.93	3.86	*D*_740_	0.91	4.51
*RII*	0.95	4.27	*D*_740_	0.90	6.25	*R*_750_/*R*_550_	0.93	3.87	*R*_750_/*R*_710_	0.88	5.22
*D*_740_	0.95	4.41	TCARI/OSAVI	0.88	6.88	*R*_860_/*R*_550_	0.93	3.88	*D*_730_	0.87	5.44
*R*_860_/*R*_550_	0.95	4.49	*D*_754_/*D*_704_	0.87	7.10	*R*_750_/*R*_700_	0.93	3.94	*R*_750_/*R*_550_	0.84	5.95
1/*R*_550_−1/*R*_750_	0.95	4.54	*D*_730_	0.86	7.51	SD: *R*_748_−*R*_747_	0.93	4.00	*R*_860_/(*R*_550_* *R*_708_)	0.84	5.95

*, the MDATT indices that performed best in each dataset. White poplar dataset, *n*=55; Chinese elm dataset, *n*=60.

The (*R*_719_ *−R*_731_)/(*R*_719_ *−R*_647_) and (*R*_719_ *−R*_746_)/(*R*_719_ *−R*_718_) MDATT indices generated significant linear relationships with LCC for adaxial (*R* ^2^=0.96, *RMSE*=3.84 μg/cm^2^) and abaxial (*R* ^2^=0.95, *RMSE*=4.26 μg/cm^2^) leaf surfaces of white poplar. Similar good relationships were also obtained for Chinese elm, where the (*R*_719_ *−R*_761_)/(*R*_719_ *−R*_493_) MDATT index gave significant linear relationships with LCC for adaxial surfaces (*R* ^2^=0.95, *RMSE*=3.51 μg/cm^2^) and the (*R*_719_ *−R*_742_)/(*R*_719_ *−R*_732_) index did so for abaxial surfaces (*R* ^2^=0.93, *RMSE*=4.07 μg/cm^2^).

In addition, the published spectral indices that achieved an equally good relationship to LCC were almost always based on red edge region wavelengths such as VOG_2_. Although most published spectral indices could give reasonable accuracy on the adaxial surface, they behaved relatively poorly on the abaxial surface. For example, the VOG_2_ gave a *R* ^2^ value of 0.96 for the adaxial surface of white poplar dataset, but only 0.90 for the abaxial surface dataset.

## Discussion

### Analysis of spectra of adaxial and abaxial leaf surfaces

The abaxial leaf surface had systematically larger visible reflectance than the adaxial surface, whatever the species (Fig. 2). However, the distinction was notable only in visible wavelengths, whereas in the near infrared the reflectance did not show an obvious difference between the two surfaces (Fig. 3). This reflectance difference exists perhaps because most leaves have a distinct layer of long palisade parenchyma tissues in the upper part of the mesophyll and more irregularly shaped, loosely arranged spongy parenchyma tissues in the lower part of the mesophyll (Gates *et al.*, 1965). The abaxial side has larger aerial interspaces between the mesophyll cells and a consequently greater extent for light reflectance processes (Cordón and Lagorio, 2007). It was also found that the reflectance at visible wavelengths for abaxial surfaces of white poplar was much higher than for Chinese elm because of the dense tubular hairs on the abaxial surface of white poplar.

Because all the VIs were derived from leaf spectra, one VI value may be altered more or less according to the difference between adaxial and abaxial spectra in one piece of leaf. Therefore, it is necessary to ensure that a VI is stable enough before using it to estimate LCC when reflectance spectra from both leaf surfaces are considered. The difference in reflectance between adaxial and abaxial surfaces in Fig. 3 indicates that the smallest change in reflectance occurred in the red edge regions (735nm for white poplar and 728nm for Chinese elm). It also suggests that robust VIs may be derived from these wavelengths or from wavelengths adjoining them. The MDATT index that was found to be least sensitive to the adaxial or abaxial surface included the 700~760nm wavelengths that were very similar to the zero-change reflectance wavelength.

### Relationships of VIs with LCC on adaxial or abaxial surfaces

When the two species were analysed separately, almost all the VIs had stronger relationships with LCC on adaxial surfaces than on abaxial surfaces because the large aerial interspaces in the spongy tissues and the dense hairs of abaxial surfaces increased reflectance and resulted in greater errors in Chl content prediction than on adaxial surfaces.

In particular, many of the published indices studied in this paper performed well when only adaxial surface data were tested (Table 3). For example, the VOG_2_ index showed a strong correlation with LCC for the adaxial surfaces in the white poplar dataset, with a high *R* ^2^ value of 0.96 and an *RMSE* of 3.90 μg/cm^2^. However, for the abaxial surfaces in the white poplar dataset, the *R* ^2^ value was 0.90 and the *RMSE* was 6.37 μg/cm^2^. The other published indices also showed the same tendency. Although the published VIs that performed well in estimating LCC measured using the SPAD chlorophyll meter were also discussed in Lu and Lu’s (2015) study, their results were not as accurate as those presented in this paper because the Chl meter included some error in measuring the LCC [the *R* ^2^ between the SPAD index and LCC was only 0.90 for white poplar and 0.85 for Chinese elm (Lu and Lu, 2015)].

It can be concluded that these indices are valid if they are used only on adaxial surfaces under exactly the same conditions for which they were designed, on the species examined in the study. However, they did not generate good results for the dataset of abaxial surfaces. The most likely reason is that the indices were developed by regression analysis between LCC and adaxial leaf surface reflectance. The observed reflectance difference between adaxial and abaxial leaf surfaces was not taken into account when the indices were developed.

### Stable VIs for predicting LCC independent of leaf species or surfaces

A helpful comparison of VI performance can be made using a dataset that mixes both adaxial and abaxial leaf surfaces. Empirical models for estimating LCC by MDATT indices for both adaxial and abaxial surfaces are largely based on the reflectance band of red edge regions (700~760nm) (Fig. 5). The figure showed that MDATT indices based on these wavelength reflectances had good applicability for LCC prediction if the reflectance from different phenotypic surfaces, such as the abaxial surfaces of white poplar leaves with their very dense hair, is considered. A possible explanation is that the reflectances are most similar between adaxial and abaxial leaf surfaces. (*R*_719_ *−R*_726_)/(*R*_719_ *−R*_743_) could be the best VI for estimating leaf Chl content, whatever the leaf side or species. The (*R*_719_ *−R*_732_)/(*R*_719_ *−R*_726_) and (*R*_719_ *−R*_747_)/(*R*_719_ *−R*_721_) indices, which are composed of very similar bands to the (*R*_719_ *−R*_726_)/(*R*_719_ *−R*_743_) index, were selected for white poplar and Chinese elm, respectively, because they possessed the strongest relationship to LCC.

Note that the MDATT indices combined with the red edge wavelengths performed better than combinations with other wavelengths between 400 and 1000nm (Figs 5, 6). The most likely reason is that the differences in reflectance between adaxial and abaxial surfaces were least within the red edge region. Datt (1999b) proposed a (*R*_850_ *−R*_710_)/(*R*_850_ *−R*_680_) index to predict the LCC of *Eucalyptus* plants. This index only generated an *R* ^2^ value of 0.73 when estimating LCC in a dataset of the two surfaces of the two species (Table 2). The selection of the 680nm and 850nm wavelengths in the red edge region, which showed relatively greater differences in reflectance between the two leaf surfaces as well as spectrometer noise at the longer wavelength (850nm), may have caused the decrease in *R* ^2^ for the Datt index. Although the results of Datt’s study showed that (*R*_850_ *−R*_710_)/(*R*_850_ *−R*_680_) was effective in estimating LCC for *Eucalyptus* plants, it was not very applicable to LCC prediction, including adaxial and abaxial reflectance information used in this study. However, the MDATT indices with a fixed *λ*_3_ at 850nm still could generate *R* ^2^ greater than 0.8 if *λ*_1_ and *λ*_2_ were set within the red edge region (Fig. 5).

Leaf reflectance (*R*) was modelled by Baret *et al.* (1988) using the following formula:

$$R=R_{s}+S\exp\left(-k_{i}C_{i}\right)$$ (5)

where *R_s_* is the reflectance at the leaf surface, *S* represents the scattering effects of the leaf mesophyll structure on reflectance, and *k*_*i*_ and *C*_*i*_ are, respectively, the specific absorption coefficient and the concentration of leaf biochemical *i* (Datt, 1999b). *R*_s_ and *S* are thought to be the main factors influencing LCC estimation variability between different samples because they depend on the differences in leaf surface and internal mesophyll structure of different samples, but do not vary between samples due to leaf biochemicals.

For the development of a specular reflectance and scatter insensitive Chl index, three wavelengths were used, for which Eq. (5) can be rewritten as follows:

$$R_{\lambda_{1}}=R_{s}+S\exp\left(-k_{chl(\lambda_{1})}C_{chl}\right)$$ (6)

$$R_{\lambda_{2}}=R_{s}+S\exp\left(-k_{chl(\lambda_{2})}C_{chl}\right)$$ (7)

$$R_{\lambda_{3}}=R_{s}+S\exp\left(-k_{chl(\lambda_{3})}C_{chl}\right)$$ (8)

where *C*_*chl*_ is the chlorophyll content and *k*_*chl*(*λ1*)_, *k*_chl(*λ2*)_ and *k*_*chl*(*λ3*)_ are the specific absorption coefficients for Chl at *λ*_*1*_, *λ*_2_ and *λ*_3_, respectively.

Taking differences between Eqs (6) and (8) and Eqs (7) and (8) and dividing the results gives:

$$\frac{\left(R_{\lambda_{3}}-R_{\lambda_{1}}\right)}{\left(R_{\lambda_{3}}-R_{\lambda_{2}}\right)}=\frac{\exp(-k_{chl(\lambda_{3})}C_{chl})-\exp(-k_{chl(\lambda_{1})}C_{chl})}{\exp(-k_{chl(\lambda_{3})}C_{chl})-\exp(-k_{chl(\lambda_{2})}C_{chl})}$$ (9)

Equation (9) is now related to Chl absorption only and is independent of the additive and multiplicative effects of leaf structure (Datt, 1999b). Therefore, the MDATT indices have removed the effects of *R*_*s*_ and *S* by taking the difference in reflectance between two wavelength bands and then taking the ratio of two such differences. Datt (1999b) developed the (*R*_850_ *−R*_710_)/(*R*_850_ *−R*_680_) index by taking advantage of the lack of absorption by leaf pigments at 850nm, although the results obtained here did not show that taking 850nm as the *λ*_3_ wavelength was effective in removing the effect of the reflectance differences between adaxial and abaxial surfaces. Taking account of the three bands within the red edge region, which correlates with LCC, was shown to be highly superior to the Datt index in predicting LCC.

The relationship of the SD VIs (*R* ^2^=0.81 for *R*_709_ *−R*_434_ for both plants, 0.83 for *R*_708_ *−R*_434_ for white poplar and 0.76 for *R*_712_ *−R*_426_ for Chinese elm, respectively) to LCC (shown in Table 2) seems to be less satisfactory compared to the MDATT indices, but better than the other two-band indices. If Eq. (5) was substituted into the SD indices, then Eq. (10) would explain the possible reasons for the poorer performance of the SD indices:

$$\begin{array}{l} SD(R_{\lambda_{1}},R_{\lambda_{2}})=R_{\lambda_{1}}-R_{\lambda_{2}}=S\exp\\ \left(-k_{chl(\lambda_{1})}C_{chl}\right)-S\exp\left(-k_{chl(\lambda_{2})}C_{chl}\right) \end{array}$$ (10)

In the SD indices, the leaf surface effect (*R*_*s*_) on the LCC estimates could be removed, but the influence of scattering from mesophyll structures (*R*_*s*_) would still remain. As for the SR and NR indices, neither the effect of *R*_*s*_ nor that of *S* was eliminated.

In addition, the scatter plots for the 230 samples between LCC and the best-performing MDATT, SD, SR and ND indices, as shown in Fig. 7, revealed that the MDATT index correlates better with LCC than the two-band indices. For example, the MDATT (*R*_719_ *−R*_726_)/(*R*_719_ *−R*_743_) index had the highest correlation with LCC (*R* ^2^=0.92), whereas the SD *R*_709_ *−R*_434_ index (*R* ^2^=0.81), the SR *R*_451_/*R*_604_ index (*R* ^2^=0.78) and the ND (*R*_583_ *−R*_426_)/(*R*_583_+*R*_426_) index (*R* ^2^=0.76) showed much lower linear *R* ^2^. Hence, caution is necessary when selecting not only applicable bands, but also suitable VI types to predict LCC.

Most of the published indices performed poorly, especially in the dataset that mixed different leaf surfaces. The *D*_754_/*D*_704_ and (*R*_850_ *−R*_710_)/(*R*_850_ *−R*_680_) indices gave the least reasonable performance among all the published indices for white poplar, Chinese elm, or both species combined, independent of the leaf surface. This occurred perhaps because all these indices also have the same form as the MDATT index. Taking *D*_754_/*D*_704_ as an example, *D*_754_ and *D*_704_ are also the reflectance differences near the 754nm and 704nm wavelengths. As a result, *D*_754_/*D*_704_ is also the type of VI that takes the ratio of two differences in reflectance between two wavelength bands, similarly to the MDATT index proposed in this paper. It might have removed some effects of *R*_*s*_ and *S*. The main distinction between *D*_754_/*D*_704_ and the MDATT index was the wavelength selection. Although *D*_754_/*D*_704_ also selected the red edge bands, it was not as effective as the MDATT index proposed in this study in estimating LCC when considering both adaxial and abaxial leaf surfaces. *D*_754_/*D*_704_ showed *R* ^2^ values of only 0.76, 0.81 and 0.72 for the two-surface dataset of the two species combined, white poplar and Chinese elm, respectively. The other published indices did not generate good results with the observed data from leaves of the two plants combined because of their limited generality for addressing different leaf structures, which may be strongly impacted by epidermis and mesophyll structures or phenotypic characteristics.

Note that the developed VIs are more generally useful and applicable to LCC prediction for leaves with structures similar to those in this study. When estimating leaves with other phenotypic characteristics, the validity of the VIs must be confirmed.

## Conclusions

Based on an analysis of the quantitative relationships between LCC and various narrow-band VIs, a new spectral index has been derived that is useful for estimating LCC in different plant species for both adaxial and abaxial surfaces. A newly developed modified Datt index, (*R*_719_ *−R*_726_)/(*R*_719_ *−R*_743_), exhibited the best performance among all the VIs tested. Newly developed indices were proposed for measuring LCC and showed improved prediction ability compared to previously published spectral indices. Therefore, the (*R*_719_ *−R*_726_)/(*R*_719_ *−R*_743_) spectral index is recommended for reliable estimation of LCC when the reflectance comes from both adaxial and abaxial leaf surfaces. This experimental study was carried out on two plant species with different phenotypic characteristics. Therefore, the results presented here include potential relationships that might be common to other plant species similar to those studied here. The research provides useful insights for phenotypic vegetation research because the accuracy of the Chl content estimation was greatly improved by removing the effects of leaf adaxial and abaxial surfaces. Further studies are needed to examine their applicability to other plant species.

## Supplementary data

Supplementary data is available at JXB online.

Supplementary Table S1. Relationships between vegetation indices and leaf chlorophyll content for combined adaxial and abaxial data of two species with different leaf surfaces.

Supplementary Table S2. Relationships between vegetation indices and leaf chlorophyll content for separate adaxial and abaxial data of two species with different leaf surfaces.

Supplementary Data

## Abbreviations:

CARI: chlorophyll absorption ratio index
Chl: chlorophyll
LCC: leaf chlorophyll content
MCARI: modified chlorophyll absorption ratio index
MDATT: modified Datt index
ND: normalized difference
NDI: normalized difference index
NIR: near infrared
PSND: pigment-specific normalized difference
PSSR: pigment-specific simple ratio
R^2^: coefficient of determination
RMSE: root mean square error
SD: simple difference
SPAD: soil plant analysis development
SR: simple ratio
TCARI: transformed chlorophyll absorption in reflectance index
TCARI/OSAVI: transformed chlorophyll absorption in reflectance index/ optimized soil-adjusted vegetation index
VI: vegetation index
VOG_2_: Vogelmann red edge index 2
R_λ_: reflectance at wavelength λ.
