# Supplementary material for: Comparing vegetation indices for remote chlorophyll measurement of white poplar and Chinese elm leaves with different adaxial and abaxial surfaces
Source: J Exp Bot. 2015 Jun 1;66(18):5625–37. doi: 10.1093/jxb/erv270 (PMC4585420; doi:10.1093/jxb/erv270)
Supplement: Supplementary Data [file supp_66_18_5625__index.html]

Comparing vegetation indices for remote chlorophyll measurement of white poplar and Chinese elm leaves with different adaxial and abaxial surfaces — Comparing vegetation indices for remote chlorophyll measurement of white poplar and Chinese elm leaves with different adaxial and abaxial surfaces — Supplementary Data 

# Comparing vegetation indices for remote chlorophyll measurement of white poplar and Chinese elm leaves with different adaxial and abaxial surfaces

## Supplementary Data

Data files

- Supplementary Data - Supplementary Data
