# Supplementary material for: Comparing vegetation indices for remote chlorophyll measurement of white poplar and Chinese elm leaves with different adaxial and abaxial surfaces
Source: J Exp Bot. 2015 Jun 1;66(18):5625–37. doi: 10.1093/jxb/erv270 (PMC4585420; doi:10.1093/jxb/erv270)
Supplement: Supplementary Data [file supp_erv270_jexbot148700_file001.pdf]

Table S1. Relationships between vegetation indices and leaf chlorophyll content for both adaxial and abaxial data of two plants with different leaf surfaces (* the MDATT indices which performed best in each dataset. The number of samples in the "both plants" dataset was 230; the number of samples in the "white poplar" dataset was 110; the number of samples in the "Chinese elm" dataset was 120).

Vegetation Indices	Both plants		Vegetation Indices	white poplar		Vegetation Indices	Chinese elm	
	R^2	*RMSE* (μg/cm^2)		R^2	*RMSE* (μg/cm^2)		R^2	*RMSE* (μg/cm^2)
MDATT: $(R_{\lambda3}-R_{\lambda1})/(R_{\lambda3}-R_{\lambda2})$ (R^2>0.92:λ_1:726~728, λ_2:743~743, λ_3:717~720) (R^2>0.90:λ_1:721~746, λ_2:705~758, λ_3:699~798) $(R_{719}-R_{726})/(R_{719}-R_{743})$ *	0.92	5.23	MDATT: $(R_{719}-R_{732})/(R_{719}-R_{726})$ *	0.94	4.67	MDATT: $(R_{719}-R_{747})/(R_{719}-R_{721})$ *	0.91	4.53
SD: $R_{\lambda1}-R_{\lambda2}$ (R^2> 0.73: λ_1:705-715, λ_2:420~440) $R_{709}-R_{434}$	0.81	7.77	SD: $R_{708}-R_{434}$	0.83	8.07	SD: $R_{712}-R_{426}$	0.76	7.35
SR: $R_{\lambda1}/R_{\lambda2}$ (R^2>0.70: λ_1:400~680, λ_2:510~702) R_{451}/R_{604}	0.78	8.51	D_{754}/D_{704}	0.81	8.66	SR: R_{421}/R_{700}	0.75	7.42

Table S1 (continued)

Vegetation Indices	Both plants		Vegetation Indices	white poplar		Vegetation Indices	Chinese elm	
	R^2	*RMSE* (μg/cm^2)		R^2	*RMSE* (μg/cm^2)		R^2	*RMSE* (μg/cm^2)
D_{754}/D_{704}	0.76	8.85	SR:R_{434}/R_{517}	0.80	8.78	ND:$(R_{700}-R_{420})/(R_{700}+R_{420})$	0.73	7.66
ND:$\|(R_{\lambda1}-R_{\lambda2})\|/(R_{\lambda1}+R_{\lambda2})$ (R^2>0.60:λ_1:515~605or 700~705, λ_2:410~435) $(R_{583}-R_{426})/(R_{583}+R_{426})$	0.76	8.86	TCARI/OSAVI	0.78	9.26	D_{754}/D_{704}	0.72	7.92
$(R_{850}-R_{710})/(R_{850}-R_{680})$	0.73	9.25	ND:$(R_{516}-R_{431})/(R_{516}+R_{431})$	0.78	9.29	$(R_{850}-R_{710})/(R_{850}-R_{680})$	0.68	8.43
TCARI/OSAVI	0.71	9.72	$(R_{850}-R_{710})/(R_{850}-R_{680})$	0.76	9.75	VOG$_2$	0.67	8.47
$R_{705}/(R_{717}+R_{491})$	0.65	10.58	$R_{705}/(R_{717}+R_{491})$	0.65	11.66	D_{740}	0.67	8.49
$R_{672}/(R_{550}*R_{708})$	0.62	11.05	MCARI	0.60	12.54	D_{730}	0.63	9.08

Table S1 (continued)

Vegetation Indices	Both plants		Vegetation Indices	white poplar		Vegetation Indices	Chinese elm	
	R^2	*RMSE* (μg/cm^2)		R^2	*RMSE* (μg/cm^2)		R^2	*RMSE* (μg/cm^2)
MCARI	0.58	11.60	TCARI	0.60	12.54	TCARI/OSAVI	0.63	9.09
TCARI	0.58	11.60	$R_{672}/(R_{550}*R_{708})$	0.59	12.66	$R_{705}/(R_{717}+R_{491})$	0.62	9.15
R_{450}/R_{550}	0.54	12.19	R_{450}/R_{550}	0.53	13.62	CARI	0.61	9.26
D_{740}	0.47	13.06	CARI	0.45	14.71	$R_{672}/(R_{550}*R_{708})$	0.61	9.27
CARI	0.44	13.38	$R_{434}/(R_{496}+R_{401})$	0.43	14.98	R_{450}/R_{550}	0.58	9.60
VOG_2	0.44	13.42	D_{740}	0.39	15.41	R_{750}/R_{710}	0.57	9.79

Table S1 (continued)

Vegetation Indices	Both plants		Vegetation Indices	white poplar		Vegetation Indices	Chinese elm	
	R^2	*RMSE* (μg/cm^2)		R^2	*RMSE* (μg/cm^2)		R^2	*RMSE* (μg/cm^2)
D_{730}	0.43	13.51	D_{730}	0.36	15.84	MCARI	0.52	10.25
R_{750}/R_{710}	0.34	14.55	VOG$_2$	0.35	15.92	TCARI	0.52	10.25
$R_{860}/(R_{550}*R_{708})$	0.30	15.00	R_{750}/R_{710}	0.24	17.24	NDI	0.51	10.42
RII	0.28	15.25	R_{672}/R_{550}	0.22	17.49	*RII*	0.47	10.83
NDI	0.27	15.30	$R_{860}/(R_{550}*R_{708})$	0.22	17.46	$R_{860}/(R_{550}*R_{708})$	0.46	10.93
R_{750}/R_{700}	0.27	15.39	*RII*	0.22	17.49	$1/R_{700}$	0.45	11.00
R_{860}/R_{550}	0.26	15.42	NDI	0.22	17.46	$1/R_{700}$-$1/R_{750}$	0.45	11.05
$1/R_{700}$-$1/R_{750}$	0.26	15.42	R_{860}/R_{550}	0.21	17.55	$1/R_{550}$-$1/R_{750}$	0.44	11.16

Table S1 (continued)

Vegetation Indices	Both plants		Vegetation Indices	white poplar		Vegetation Indices	Chinese elm	
	R^2	*RMSE* (μg/cm^2)		R^2	*RMSE* (μg/cm^2)		R^2	*RMSE* (μg/cm^2)
R_{750}/R_{550}	0.26	15.44	R_{750}/R_{700}	0.21	17.58	R_{750}/R_{700}	0.42	11.27
$1/R_{550}-1/R_{750}$	0.26	15.48	R_{750}/R_{550}	0.21	17.60	R_{750}/R_{550}	0.42	11.35
$1/R_{700}$	0.25	15.59	$1/R_{700}-1/R_{750}$	0.20	17.66	R_{860}/R_{550}	0.41	11.40
R_{672}/R_{550}	0.24	15.62	$1/R_{550}-1/R_{750}$	0.20	17.71	PSSR$_b$	0.31	12.37
$R_{434}/(R_{496}+R_{401})$	0.21	15.99	$1/R_{700}$	0.19	17.84	$(R_{800}-R_{635})/(R_{800}+R_{635})$	0.29	12.50
PSSR$_b$:R_{800}/R_{635}	0.19	16.15	PSSR$_b$	0.15	18.27	R_{800}/R_{650}	0.23	13.01
R_{800}/R_{650}	0.14	16.62	R_{800}/R_{650}	0.11	18.64	$(R_{800}-R_{650})/(R_{800}+R_{650})$	0.23	13.06
$(R_{800}-R_{635})/(R_{800}+R_{635})$	0.07	17.28	$(R_{800}-R_{635})/(R_{800}+R_{635})$	0.06	19.17	R_{680}	0.14	13.82
R_{800}/R_{675}	0.06	17.37	D_{710}	0.05	19.27	$(R_{800}-R_{675})/(R_{800}+R_{675})$	0.14	13.82

Table S1 (continued)

Vegetation Indices	Both plants		Vegetation Indices	white poplar		Vegetation Indices	Chinese elm	
	R^2	*RMSE* (μg/cm^2)		R^2	*RMSE* (μg/cm^2)		R^2	*RMSE* (μg/cm^2)
$PSSR_a$	0.06	17.38	R_{950}/R_{680}	0.05	19.29	R_{800}/R_{675}	0.13	13.87
R_{950}/R_{680}	0.06	17.39	$PSSR_a$	0.05	19.30	D_{710}	0.13	13.88
D_{710}	0.04	17.58	R_{800}/R_{675}	0.05	19.30	$PSSR_a$	0.12	13.91
$(R_{800}-R_{650})/(R_{800}+R_{650})$	0.03	17.66	$(R_{800}-R_{650})/(R_{800}+R_{650})$	0.03	19.49	R_{950}/R_{680}	0.12	13.95
R_{680}	0.01	17.84	R_{680}	0.01	19.61	R_{672}/R_{550}	0.09	14.18
$(R_{800}-R_{675})/(R_{800}+R_{675})$	0.00	17.96	$(R_{800}-R_{675})/(R_{800}+R_{675})$	0.00	19.76	$R_{434}/(R_{496}+R_{401})$	0.06	14.44

Table S2. Relationships between vegetation indices and leaf chlorophyll content for individual plant species (* the MDATT indices which performed best in each dataset. The number of samples in the "white poplar" dataset was 55; the number of samples in the "Chinese elm" dataset was 60).

	white poplar						Chinese elm				
	Adaxial surface			Abaxial surface			Adaxial surface			Abaxial surface	
Vegetation Indices	R^2	*RMSE* (μg/cm^2)	Vegetation Indices	R^2	*RMSE* (μg/cm^2)	Vegetation Indices	R^2	*RMSE* (μg/cm^2)	Vegetation Indices	R^2	*RMSE* (μg/cm^2)
MDATT: $(R_{\lambda3}-R_{\lambda1})/(R_{\lambda3}-R_{\lambda2})$<br>($R^2$>0.96:$\lambda_1$:715~751, λ_2:646~739, λ_3:400~788)<br>$(R_{719}-R_{731})/(R_{719}-R_{647})$*	0.96	3.84	MDATT: $(R_{719}-R_{746})/(R_{719}-R_{718})$ *	0.95	4.26	MDATT: $(R_{719}-R_{761})/(R_{719}-R_{493})$ *	0.95	3.51	MDATT: $(R_{719}-R_{742})/(R_{719}-R_{732})$ *	0.93	4.07
SR:$R_{\lambda1}/R_{\lambda2}$<br>($R^2$>0.83:$\lambda_1$:740~780, λ_2:700~750)<br>R_{751}/R_{720}	0.96	3.78	SR: R_{747}/R_{748}	0.92	5.52	SR:R_{779}/R_{709}	0.95	3.48	SR:R_{742}/R_{739}	0.92	4.17
ND:$\|(R_{\lambda1}-R_{\lambda2})\|/(R_{\lambda1}+R_{\lambda2})$<br>($R^2$>0.94:$\lambda_1$:740~750, λ_2:730~750)<br>$(R_{740}-R_{738})/(R_{740}+R_{738})$	0.96	3.83	ND:$(R_{748}-R_{747})/(R_{748}+R_{747})$	0.92	5.52	R_{750}/R_{710}	0.95	3.51	ND:$\|(R_{742}-R_{739})\|/(R_{742}+R_{739})$	0.92	4.19
R_{750}/R_{710}	0.96	3.83	SD: $R_{748}-R_{747}$	0.92	5.6	VOG_2	0.94	3.54	VOG_2	0.92	4.21

Table S2 (continued)

Vegetation Indices	white poplar Adaxial surface R^2	Adaxial surface $RMSE$ (μg/cm^2)	Vegetation Indices	Abaxial surface R^2	Abaxial surface $RMSE$ (μg/cm^2)	Chinese elm Vegetation Indices	Adaxial surface R^2	Adaxial surface $RMSE$ (μg/cm^2)	Vegetation Indices	Abaxial surface R^2	Abaxial surface $RMSE$ (μg/cm^2)
VOG_2	0.96	3.90	$(R_{850}-R_{710})/(R_{850}-R_{680})$	0.91	5.86	ND:$(R_{749}-R_{738})/(R_{749}+R_{738})$	0.94	3.57	SD:$R_{749}-R_{746}$	0.91	4.45
SD:$R_{\lambda1}-R_{\lambda2}$ (R^2>0.94:λ_1:740~750, λ_2:740~750) $R_{744}-R_{742}$	0.95	4.30	VOG_2	0.90	6.37	*RII*	0.93	3.86	D_{740}	0.91	4.51
RII	0.95	4.27	D_{740}	0.90	6.25	R_{750}/R_{550}	0.93	3.87	R_{750}/R_{710}	0.88	5.22
D_{740}	0.95	4.41	TCARI/OSAVI	0.88	6.88	R_{860}/R_{550}	0.93	3.88	D_{730}	0.87	5.44
R_{860}/R_{550}	0.95	4.49	D_{754}/D_{704}	0.87	7.10	R_{750}/R_{700}	0.93	3.94	R_{750}/R_{550}	0.84	5.95
$1/R_{550}-1/R_{750}$	0.95	4.54	D_{730}	0.86	7.51	SD:$R_{748}-R_{747}$	0.93	4.00	$R_{860}/(R_{550}*R_{708})$	0.84	5.95

Table S2 (continued)

Vegetation Indices	white poplar		Vegetation Indices			Vegetation Indices	Chinese elm		Vegetation Indices		
	Adaxial surface			Abaxial surface			Adaxial surface			Abaxial surface	
	R^2	*RMSE* (μg/cm^2)		R^2	*RMSE* (μg/cm^2)		R^2	*RMSE* (μg/cm^2)		R^2	*RMSE* (μg/cm^2)
R_{750}/R_{550}	0.95	4.57	R_{450}/R_{550}	0.77	9.58	$1/R_{700}$-$1/R_{750}$	0.93	4.06	R_{860}/R_{550}	0.84	5.96
$1/R_{700}$-$1/R_{750}$	0.94	4.64	TCARI	0.75	9.93	D_{740}	0.92	4.22	D_{754}/D_{704}	0.84	6.00
$1/R_{700}$	0.94	4.71	MCARI	0.75	9.93	$1/R_{700}$	0.92	4.29	$(R_{850}$-$R_{710})/(R_{850}$ -$R_{680})$	0.83	6.26
R_{750}/R_{700}	0.94	4.74	$R_{705}/(R_{717}+R_{491})$	0.75	9.92	$1/R_{550}$-$1/R_{750}$	0.92	4.30	$1/R_{550}$-$1/R_{750}$	0.82	6.36
$R_{860}/(R_{550}*R_{708})$	0.94	5.04	$R_{672}/(R_{550}*R_{708})$	0.72	10.57	$R_{860}/(R_{550}*R_{708})$	0.91	4.54	R_{750}/R_{700}	0.82	6.37
D_{730}	0.93	5.26	R_{750}/R_{710}	0.71	10.70	D_{730}	0.88	5.14	*RII*	0.82	6.38
NDI	0.92	5.58	R_{672}/R_{550}	0.69	11.16	$R_{672}/(R_{550}*R_{708})$	0.87	5.48	NDI	0.81	6.49

Table S2 (continued)

Vegetation Indices	white poplar Adaxial surface R^2	white poplar Adaxial surface $RMSE$ (μg/cm^2)	Vegetation Indices	white poplar Abaxial surface R^2	white poplar Abaxial surface $RMSE$ (μg/cm^2)	Vegetation Indices	Chinese elm Adaxial surface R^2	Chinese elm Adaxial surface $RMSE$ (μg/cm^2)	Vegetation Indices	Chinese elm Abaxial surface R^2	Chinese elm Abaxial surface $RMSE$ (μg/cm^2)
$(R_{850}-R_{710})/(R_{850}-R_{680})$	0.92	5.63	$R_{434}/(R_{496}+R_{401})$	0.63	12.14	NDI	0.86	5.59	$1/R_{700}-1/R_{750}$	0.81	6.57
CARI	0.91	6.14	NDI	0.54	13.51	D_{754}/D_{704}	0.86	5.64	CARI	0.78	6.95
$R_{705}/(R_{717}+R_{491})$	0.90	6.19	CARI	0.53	13.68	R_{450}/R_{550}	0.85	5.82	PSSR$_b$	0.78	7.04
TCARI/OSAVI	0.89	6.61	R_{680}	0.52	13.77	$(R_{850}-R_{710})/(R_{850}-R_{680})$	0.84	5.93	$1/R_{700}$	0.77	7.24
R_{450}/R_{550}	0.89	6.66	$(R_{800}-R_{675})/(R_{800}+R_{675})$	0.44	14.94	PSSR$_b$	0.84	5.97	$R_{705}/(R_{717}+R_{491})$	0.76	7.39
MCARI	0.88	6.81	R_{950}/R_{680}	0.42	15.22	$R_{705}/(R_{717}+R_{491})$	0.82	6.44	TCARI/OSAVI	0.75	7.52
TCARI	0.88	6.81	PSSR$_a$	0.42	15.21	CARI	0.76	7.36	R_{800}/R_{650}	0.72	7.93
$R_{672}/(R_{550}*R_{708})$	0.88	6.89	R_{800}/R_{675}	0.42	15.23	R_{800}/R_{650}	0.75	7.56	R_{450}/R_{550}	0.72	7.98

Table S2 (continued)

Vegetation Indices	white poplar Adaxial surface R^2	white poplar Adaxial surface *RMSE* (μg/cm^2)	Vegetation Indices	white poplar Abaxial surface R^2	white poplar Abaxial surface *RMSE* (μg/cm^2)	Vegetation Indices	Chinese elm Adaxial surface R^2	Chinese elm Adaxial surface *RMSE* (μg/cm^2)	Vegetation Indices	Chinese elm Abaxial surface R^2	Chinese elm Abaxial surface *RMSE* (μg/cm^2)
D_{754}/D_{704}	0.88	7.01	*RII*	0.31	16.52	MCARI	0.73	7.78	$(R_{800}-R_{635})/(R_{800}+R_{635})$	0.70	8.25
PSSR$_b$	0.83	8.33	R_{860}/R_{550}	0.27	17.03	TCARI	0.73	7.78	$R_{672}/(R_{550}*R_{708})$	0.63	9.08
$R_{434}/(R_{496}+R_{401})$	0.76	9.74	R_{750}/R_{550}	0.21	17.68	TCARI/OSAVI	0.70	8.21	$(R_{800}-R_{650})/(R_{800}+R_{650})$	0.63	9.09
$(R_{800}-R_{635})/(R_{800}+R_{635})$	0.74	10.09	R_{750}/R_{700}	0.21	17.68	$(R_{800}-R_{635})/(R_{800}+R_{635})$	0.63	9.13	R_{800}/R_{675}	0.62	9.18
R_{800}/R_{650}	0.74	10.11	R_{800}/R_{650}	0.16	18.27	R_{672}/R_{550}	0.60	9.47	PSSR$_a$	0.62	9.26
R_{672}/R_{550}	0.73	10.35	$(R_{800}-R_{650})/(R_{800}+R_{650})$	0.14	18.53	R_{800}/R_{675}	0.55	10.05	R_{950}/R_{680}	0.60	9.44
$(R_{800}-R_{650})/(R_{800}+R_{650})$	0.65	11.83	$1/R_{700}$	0.09	18.97	$(R_{800}-R_{650})/(R_{800}+R_{650})$	0.54	10.14	MCARI	0.59	9.59
R_{950}/R_{680}	0.61	12.49	$1/R_{700}-1/R_{750}$	0.04	19.57	PSSR$_a$	0.53	10.24	TCARI	0.59	9.59

Table S2 (continued)

	white poplar						Chinese elm					
Vegetation Indices	Adaxial surface		Vegetation Indices	Abaxial surface		Vegetation Indices	Adaxial surface		Vegetation Indices	Abaxial surface	
	R^2	*RMSE* (μg/cm^2)		R^2	*RMSE* (μg/cm^2)		R^2	*RMSE* (μg/cm^2)		R^2	*RMSE* (μg/cm^2)
PSSR$_a$	0.60	12.66	PSSR$_b$	0.04	19.55	R_{950}/R_{680}	0.52	10.37	$(R_{800}-R_{675})/(R_{800}+R_{675})$	0.54	10.18
R_{800}/R_{675}	0.60	12.69	$R_{860}/(R_{550}*R_{708})$	0.03	19.66	$(R_{800}-R_{675})/(R_{800}+R_{675})$	0.43	11.28	R_{680}	0.47	10.95
$(R_{800}-R_{675})/(R_{800}+R_{675})$	0.57	13.05	$(R_{800}-R_{635})/(R_{800}+R_{635})$	0.02	19.73	R_{680}	0.39	11.71	D_{710}	0.38	11.75
R_{680}	0.47	14.54	$1/R_{550}-1/R_{750}$	0.01	19.87	D_{710}	0.19	13.45	R_{672}/R_{550}	0.02	14.82
D_{710}	0.44	14.90	D_{710}	0.01	19.82	$R_{434}/(R_{496}+R_{401})$	0.19	13.49	$R_{434}/(R_{496}+R_{401})$	0.00	14.98
